# A case of rare fungal keratitis caused by *Pseudoshiraia conidialis*

**DOI:** 10.1097/MD.0000000000044001

**Published:** 2025-08-22

**Authors:** Gui Ying, Haiqiong Sun, Haibo Xu, Long Cai, Hui Wei

**Affiliations:** a Clinical Laboratory Center, Hangzhou Red Cross Hospital, Hangzhou, Zhejiang Province, China.

**Keywords:** case report, keratitis, *Pseudoshiraia conidialis*

## Abstract

**Rationale:**

*Pseudoshiraia conidialis* is a filamentous fungus that belongs to the Shiraiaceae family. We report a rare case of fungal keratitis caused by *P conidialis*.

**Patient concerns:**

A 61-year-old female accidentally scratched her right eye with a bamboo branch, presenting with conjunctival congestion in the right eye, corneal ulcer with edema endothelial folds, anterior chamber exudation hypopyon, and lens opacity.

**Diagnoses:**

The patient’s eyes were evaluated using slit-lamp examination, fundus examination, and pus culture, and fungal keratitis was considered as the diagnosis. After culturing, the keratitis pathogen was confirmed to be *P conidialis* through rRNA gene internal transcribed spacer amplification and sequencing.

**Interventions:**

The patient underwent debridement of the cornea and was treated with a combination of tropicamide eye drops and oral itraconazole hydrochloride capsules.

**Outcomes:**

The patient showed significant improvement early in the treatment, but was not cured. Later, the patient was transferred to another hospital and was lost to follow-up.

**Lessons:**

We report the first case of fungal keratitis caused by *P conidialis*. Microbial culture remains the gold standard for diagnosing fungal keratitis. This case provides a reference for the clinical treatment of fungal keratitis caused by *P conidialis.*

## 1. Introduction

Fungal keratitis is a highly destructive corneal infection. It typically has a slow onset and often presents with subacute symptoms such as eye redness, pain, and tearing. It later develops into ulcers and blurred vision, and severe cases can lead to intraocular infection and vision loss. Over 95% of corneal fungal infections are caused by filamentous fungi such as Fusarium, Aspergillus, and yeast. Filamentous fungi are the primary cause of most fungal infections in tropical and subtropical regions, whereas yeast are more common in temperate regions.^[1]^ So far, fungal keratitis caused by *Pseudoshiraia conidialis* has not been reported. There were no related clinical findings, fungal species information, or any details of effective treatments. In this report, we describe a case of fungal keratitis caused by *P conidialis*.

## 2. Case description

**Figure 1.**
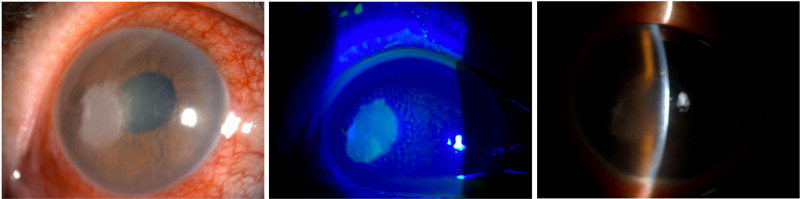
The corneal lesions in the right eye under the slit-lamp microscope before treatment.

The patient was a 61-year-old female, accidentally scratched in the right eye by a bamboo branch 1 month previously, who felt that the symptoms were not severe and did not seek any treatment. The patient visited the ophthalmology department of our hospital, where it was found that the right eye had conjunctival congestion, a round corneal ulcer with unclear borders, endothelial folds, edema, a large amount of exudate in the anterior chamber, pus accumulation in the inferior anterior chamber, a round pupil, and lens opacity (Fig. 1). The initial diagnosis was considered to be fungal keratitis. Surgical debridement of the corneal ulcer was performed immediately and pus was drained for pathogen culture. Postoperatively, the patient was treated with compound tropicamide eye drops and itraconazole hydrochloride capsules. Three days after antifungal treatment, the corneal ulcer shrank, its borders became clearer, necrotic material decreased, exudate in the anterior chamber significantly reduced, and pus in the anterior chamber was absorbed.

**Figure 2.**
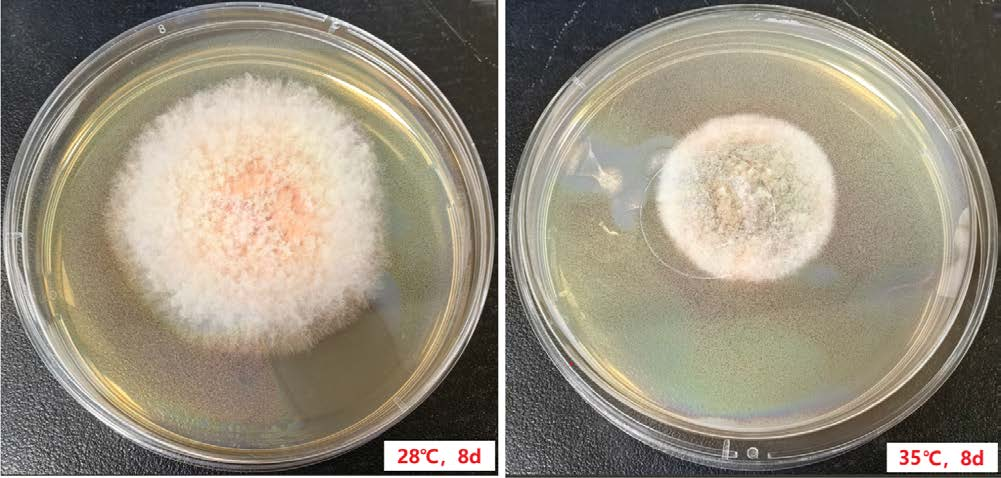
The strain grows better at 28℃ than at 35℃.

**Figure 3.**
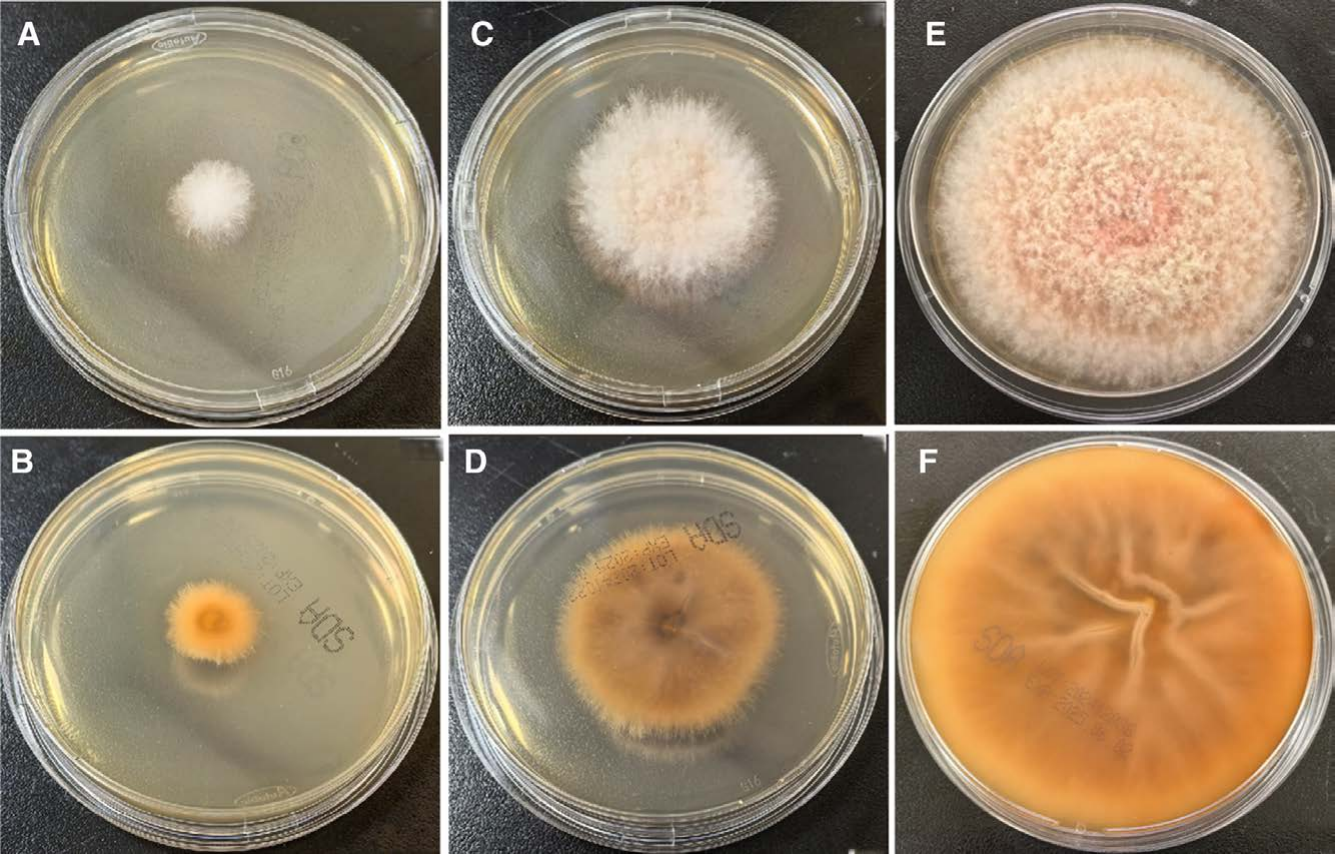
The colony morphology of (A, B) after incubation for 3 d at 28℃ on Sabouraud dextrose agar. The colony morphology of (C, D) after incubation for 7 d at 28℃ on Sabouraud dextrose agar. The colony morphology of (E, F) after incubation for 14 d at 28℃ on Sabouraud dextrose agar. Figures A, C, and E are the front side, and B, D, and F are the back side.

The pus specimen drained from the patient was directly examined under a microscope using 10% KOH, revealing a small number of fungal hyphae. The specimen was then cultured on Columbia blood agar plates and Sabouraud dextrose agar (SDA) plates, the blood agar plate was incubated at 35°C with 5% CO_2_, while the 2 SDA plates were incubated at 35°C and 28°C, respectively. The fungal strain grew better at 28°C than at 35°C (Fig. 2). After 3 days of incubation on SDA agar, the fingernail began to grow, with the surface covered by a sparse white aerial mycelium. The underside of the colonies was light orange, with the orange color gradually extending outward from the center of the inoculation site (Fig. 3A, B). After 7 days of incubation, the colonies appeared woolly or tufted in texture (Fig. 3C, D). After 14 days of incubation, the colonies spread to cover the entire plate (Fig. 3E, F). The color of the colonies on the back of the agar gradually deepened over time from light orange to dark orange, and the bottom of the agar exhibited obvious wrinkling.

**Figure 4.**
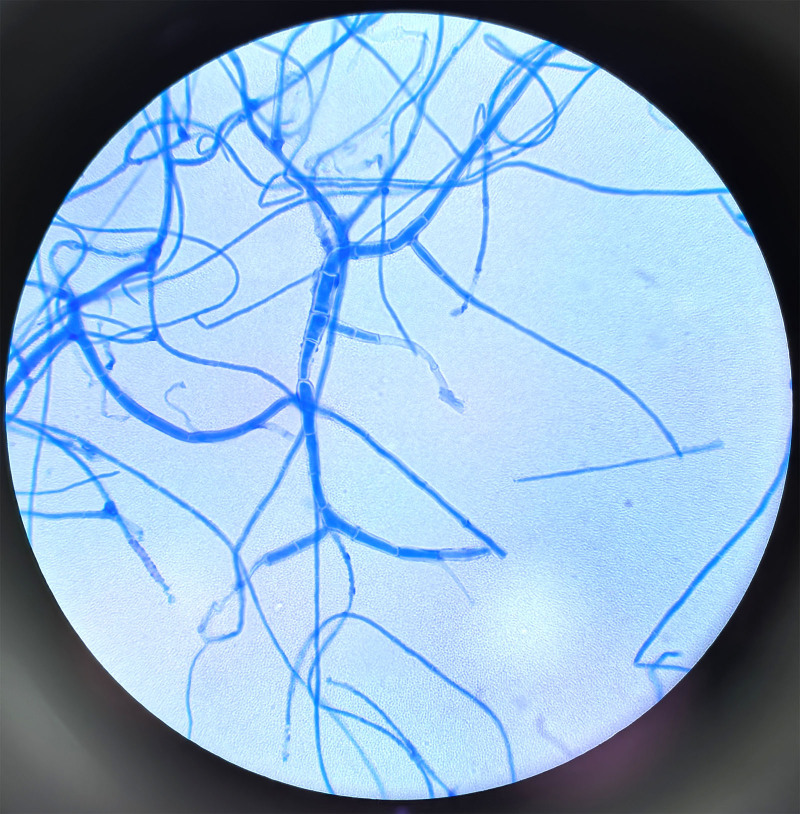
The morphology under lactic acid phenol cotton blue staining after incubation for 14 d at 28℃, ×400 magnification.

Morphological examination using lactophenol cotton blue staining revealed numerous filamentous hyphae, some of which were septate (Fig. 4).

**Figure 5.**
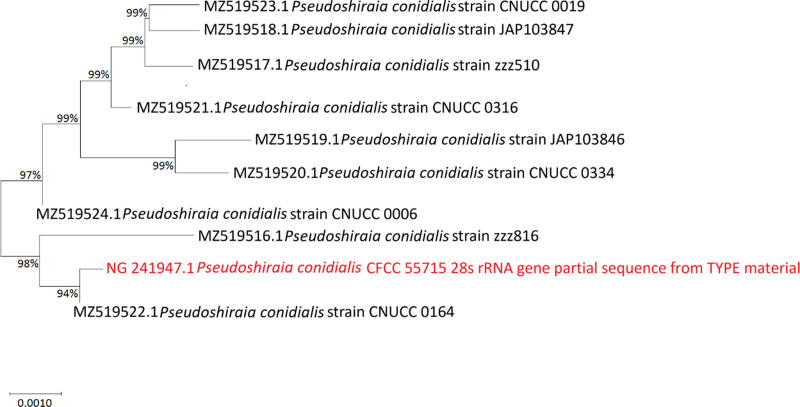
Phylogenetic tree of *Pseudoshiraia conidialis* based on ITS sequence. ITS = internal transcribed spacer.

The VITEK MS microbial identification system was unable to identify this strain, the strain was identified through rRNA gene internal transcribed spacer amplification and sequencing, after performing BLAST comparison in the GenBank database, it was found that the strain had the highest similarity (99.82%) with *P conidialis*, with accession number NG_241947.1. The strain was ultimately identified as *P conidialis*. Simultaneously, we searched for the internal transcribed spacer sequence of *P conidialis*-type strain O in the NCBI database and constructed a phylogenetic tree. The strain clustered within the standard strain group of *P conidialis* in the phylogenetic tree and showed a high degree of genetic relatedness to other standard strains, confirming that the strain was *P conidialis* (Fig. 5).

In vitro antimicrobial susceptibility testing of the strain was performed using a fungal susceptibility test kit (culture method) produced by Changsha Zhongsheng Zhongjie Biotechnology Co._,_ Ltd. Gently collect the conidia and suspend them in a 0.01% to 0.1% Tween 20 solution to prepare a suspension with a turbidity of 0.5 McFarland units, then inoculate the fungal susceptibility test plate. The fungal susceptibility test plate with added samples was placed in a fully automated microbiological susceptibility analyzer. The instrument reads the minimum inhibitory concentration values based on the interpretive standards from the Clinical and Laboratory Standards Institute and the European Committee on Antimicrobial Susceptibility Testing and provides the corresponding results for susceptibility (S), intermediate (I), dose-dependent susceptibility, and resistance (R). Candida krusei American Type Culture Collection 6258 was used as the quality control strain. The antifungal susceptibility results were as follows: Amphotericin B ≥ 16 μg/mL, itraconazole ≥ 8 μg/mL, posaconazole ≥ 2 μg/mL, voriconazole ≥ 8 μg/mL, anidulafungin ≥ 16 μg/mL, caspofungin 2 μg/mL, fluconazole ≥ 256 μg/mL, 5-fluorocytosine 8 μg/mL, and micafungin ≥ 32 μg/mL.

## 3. Discussion

Fungal keratitis is associated with severe clinical symptoms and its prognosis is worse than that of bacterial keratitis.^[2,3]^ It is an infection that can threaten vision, and is associated with poor prognosis and economic burden.^[4,5]^ Fungal keratitis, especially filamentous fungal keratitis, typically occurs in patients with a history of plant-related ocular trauma.^[6,7]^ In previous studies, Xie et al summarized 654 cases, of which 25.7% had a history of vegetative corneal trauma.^[8]^ In another study, corneal injuries from plants were reported as a major risk factor for fungal keratitis.^[2]^ Many cases of fungal keratitis have poor outcomes, ^[9,10]^ although about half of the cases show improvement in vision after treatment, and eye condition at the time of the visit was the most important predictive factor for poor outcomes. Implementing effective early intervention can prevent the condition from worsening to an irreversible stage.^[11]^

*P conidialis* is a filamentous fungus belonging to the Shiraiaceae family that is commonly found in living bamboo stalks.^[12]^ Currently, there have been no reports of human infections caused by fungi from the Shiraiaceae family. This is the first reported case of *P conidialis* from the Shiraiaceae family, which infects the human cornea. In our case, the initial diagnosis of fungal keratitis was suspected based on slit-lamp photographs, which was later confirmed through culture and sequencing as fungal keratitis caused by *P conidialis*.

In this case, the in vitro drug susceptibility test for *P conidialis* showed an minimum inhibitory concentration of >=≥8 μg/mL for itraconazole. The drug susceptibility test kit instructions did not provide a clear turning point for this strain, and 8 μg/mL was the highest itraconazole concentration on the susceptibility plate. Referring to the breakpoints for Aspergillus species, the interpretation was as follows: itraconazole > 2 μg/mL (R), amphotericin B > 2 μg/mL (R), posaconazole > 0.25 μg/mL (R), voriconazole > 2 μg/mL (R). Based on these criteria, *P conidialis* is resistant to itraconazole, amphotericin B, posaconazole, and voriconazole.

The patient underwent corneal debridement and antifungal treatment. After 3 days of oral itraconazole hydrochloride capsule treatment, the patient’s condition showed significant improvement. However, in the subsequent treatment, the patient’s condition fluctuated, showing improvement at times but worsening at other times; the treatment did not yield significant results. The patient was later transferred to another hospital and was lost to follow-up. It has been reported that itraconazole shows a “good response” in the treatment of fungal keratitis, and no significant adverse reactions have been reported in patients receiving oral itraconazole.^[13]^ Although itraconazole exhibits strong antifungal effects, its clinical application is often limited by its drug resistance.^[10]^ It is possible that the patient’s fluctuating condition is due to drug resistance, which could explain the lack of consistent improvement despite treatment.

## 4. Conclusion

Keratitis caused by *P conidialis* is rare. The clinical symptoms and slit-lamp examination findings were consistent with the characteristics of fungal keratitis, and the typical clinical symptoms, slit-lamp images, and culture sequencing results definitively diagnosed the fungal keratitis. Since *P conidialis* is a rare pathogen, there are no established drug susceptibility guidelines, and no standardized treatment protocols are available. Although oral treatment with itraconazole hydrochloride capsules resulted in significant improvement in the early stages of this case, the infection was not cured, possibly because of drug resistance. One limitation of this report was that the patient was lost to follow-up; therefore, there was no adequate tracking or review of the patient’s subsequent treatment and recovery.

## Acknowledgments

We greatly appreciate the provision of strains and clinical samples by the Microbiology Laboratory at the Hangzhou Red Cross Hospital.

## Author contributions

**Conceptualization:** Hui Wei, Gui Ying, Haiqiong Sun, Haibo Xu, Long Cai.

**Data curation:** Hui Wei, Gui Ying, Haiqiong Sun, Haibo Xu.

**Formal analysis:** Hui Wei, Gui Ying, Haiqiong Sun, Haibo Xu, Long Cai.

**Funding acquisition:** Hui Wei.

**Investigation:** Hui Wei, Gui Ying, Haiqiong Sun, Haibo Xu, Long Cai.

**Methodology:** Hui Wei, Gui Ying, Haiqiong Sun, Haibo Xu, Long Cai.

**Project administration:** Hui Wei.

**Resources:** Hui Wei, Gui Ying, Haiqiong Sun, Haibo Xu, Long Cai.

**Software:** Hui Wei, Gui Ying, Haiqiong Sun, Haibo Xu.

**Supervision:** Hui Wei, Gui Ying, Long Cai.

**Validation:** Hui Wei, Gui Ying, Haiqiong Sun, Haibo Xu, Long Cai.

**Visualization:** Gui Ying, Haiqiong Sun, Haibo Xu.

**Writing – original draft:** Hui Wei, Gui Ying, Haiqiong Sun.

**Writing – review & editing:** Hui Wei, Gui Ying, Haiqiong Sun.

## References

[1] Brown L Leck AK Gichangi M Burton MJ Denning DW. The global incidence and diagnosis of fungal keratitis. Lancet Infect Dis. 2021;21:e49–57. 33645500 10.1016/S1473-3099(20)30448-5

[2] Nayel AA Hamdy NA Massoud TH Mohamed NM. A comparison of antimicrobial regimen outcomes and antibiogram development in microbial keratitis: a prospective cohort study in Alexandria, Egypt. Graefes Arch Clin Exp Ophthalmol. 2024;262:1865–82. 38240778 10.1007/s00417-023-06362-0 PMC11106157

[3] Bourcier T Sauer A Dory A Denis J Sabou M. Fungal keratitis. J Fr Ophtalmol. 2017;40:e307–13. 28987448 10.1016/j.jfo.2017.08.001

[4] Ghenciu LA Faur AC Bolintineanu SL Salavat MC Maghiari AL. Recent advances in diagnosis and treatment approaches in fungal keratitis: a narrative review. Microorganisms. 2024;12 :161. 38257986 10.3390/microorganisms12010161 PMC10820712

[5] Huang Y Yu J Peng Q. Fungal keratitis treated with a combination of traditional Chinese medicine and Western medicine: a case report. Medicine (Baltim). 2022;101:e31976. 10.1097/MD.0000000000031976 PMC9726375 36482581

[6] Shen Z Zhang Y Li F Zhang Q. Case report: a rare fungal keratitis caused by plectosphaerella cucumerina. Ocul Immunol Inflamm. 2023;31:631–4. 35394848 10.1080/09273948.2022.2039213

[7] Hoffman JJ Burton MJ Leck A. Mycotic keratitis-a global threat from the filamentous fungi. J Fungi (Basel). 2021;7 :273. 33916767 10.3390/jof7040273 PMC8066744

[8] Xie L Zhong W Shi W Sun S. Spectrum of fungal keratitis in North China. Ophthalmology. 2006;113:1943–8. 16935335 10.1016/j.ophtha.2006.05.035

[9] Arboleda A Ta CN. Overview of mycotic keratitis. Cornea. 2024;43:1065–71. 39102310 10.1097/ICO.0000000000003559 PMC11300963

[10] Bisen AC Sanap SN Agrawal S. Etiopathology, epidemiology, diagnosis, and treatment of fungal keratitis. ACS Infect Dis. 2024;10:2356–80. 38847789 10.1021/acsinfecdis.4c00203

[11] Arunga S Kintoki GM Mwesigye J. Epidemiology of microbial keratitis in Uganda: a cohort study. Ophthalmic Epidemiol. 2020;27:121–31. 31830848 10.1080/09286586.2019.1700533 PMC7446037

[12] Tong X Wang QT Shen XY Hou CL Cannon PF. Phylogenetic position of Shiraia-like endophytes on bamboos and the diverse biosynthesis of hypocrellin and hypocrellin derivatives. J Fungi (Basel). 2021;7 :563. 34356942 10.3390/jof7070563 PMC8304798

[13] FlorCruz NV Evans JR. Medical interventions for fungal keratitis. Cochrane Database Syst Rev. 2015;2015:CD004241. 25855311 10.1002/14651858.CD004241.pub4 PMC10907972

